# Chloroplast Protein 12 Expression Alters Growth and Chilling Tolerance in Tropical Forage *Stylosanthes guianensis* (Aublet) Sw

**DOI:** 10.3389/fpls.2018.01319

**Published:** 2018-09-06

**Authors:** Kailong Li, Hong Qiu, Min Zhou, Yang Lin, Zhenfei Guo, Shaoyun Lu

**Affiliations:** ^1^State Key Laboratory for Conservation and Utilization of Subtropical Agro-bioresources, Guangdong Grassland Science Engineering Research Center, College of Life Sciences, South China Agricultural University, Guangzhou, China; ^2^College of Grassland Science, Nanjing Agricultural University, Nanjing, China

**Keywords:** chilling tolerance, CP12, GAPDH, photosynthesis, PRK, *S. guianensis*, transgenic plants

## Abstract

*Stylosanthes guianensis* (Aublet) Sw. is a tropical forage legume with soil acidity tolerance and excellent adaptation to infertile soils, but sensitive to chilling. To understand the molecular responses of *S. guianensis* to chilling, differentially expressed genes between a chilling tolerant mutant 7–1 and the wild type were identified using suppression subtractive hybridization, and eight of them were confirmed and the regulation pattern were analyzed using quantitative reverse transcription PCR (qRT-PCR). Chloroplast protein 12 (CP12) functions to regulate the Calvin cycle by forming a ternary complex with glyceraldehyde 3-phosphate dehydrogenase (GAPDH) and phosphoribulokinase (PRK). *SgCP12* transcript was induced by chilling in both plants, and higher levels were observed in 7–1 than in the wild type, implying a potential role of *SgCP12* in chilling tolerance. To confirm this, transgenic *S. guianensis* plants over-expressing or down-regulating *SgCP12* were generated, respectively. Higher *F*_v_/*F*_m_ and survival rate and lower ion leakage were observed in transgenic plants overexpressing *SgCP12* as compared with the wild type after chilling treatment, while lower *F*_v_/*F*_m_ and survival rate and higher ion leakage were found in *SgCP12* antisense plants. *SgCP12* overexpression plants showed promoted growth with increased plant height and fresh weight, while the antisense plants exhibited reduced growth with decreased plant height and fresh weight as compared with the wild type. The results indicated that regulation of *SgCP12* expression alters plant growth and chilling tolerance in *S. guianensis*. In addition, higher levels of net photosynthetic rate (*P*_n_), GAPDH and PRK activities were observed in *SgCP12* overexpression transgenic plants, while lower levels in antisense plants than in the wild type under both control and chilling conditions, indicating that altered activities of GAPDH and PRK were associated with the changed *P*_n_ in transgenic *S. guianensis*. Our results suggest that SgCP12 regulates GAPDH and PRK activities, *P*_n_, and chilling tolerance in *S. guianensis*.

## Introduction

Temperature is one of the major abiotic stress influencing plant growth and development. Perennial temperate plants can be survival under freezing temperatures through a mechanism called cold acclimation, while tropical plants is damaged or even dead after exposing to chilling conditions due to the lack of cold acclimation mechanism. Thousands of genes are reprogrammed in expression and multiple metabolic pathways are modified in both temperate and tropical plants in response to low temperature (Fowler and Thomashow, 2002; Krasensky and Jonak, 2012; Zhang et al., 2012). The Calvin–Benson cycle enzymes are more sensitive to low temperature than photochemical reactions, which reduces the utilization of light energy absorbed by chlorophyll and results in an elevated production of reactive oxygen species (ROS) (Allen and Ort, 2001; Alam and Jacob, 2002). The accumulated ROS may induce gene expression as a second messenger in signal transduction, or result in oxidative damages to photosynthetic apparatus and even lead to plant death if ROS could not be effectively scavenged (Foyer and Noctor, 2009).

*Stylosanthes guianensis* (Aublet) Sw. is a tropical forage legume with soil acidity tolerance and excellent adaptation to infertile soils (Jiang et al., 2005). It is commonly used as a cover crop in southern China as well. Unfortunately it is sensitive to chilling. Our previous investigations using growth chamber showed that *S. guianensis* seedlings were damaged upon exposure to low temperature at 10°C (Zhou et al., 2005a). Application of abscisic acid (ABA) increased its chilling tolerance through induced antioxidant defense system (Zhou et al., 2005a) and protected photosystem II (PSII) of photosynthesis (Zhou et al., 2006), while nitric oxide (NO) and Ca^2+^ are involved in ABA induced antioxidant defense (Zhou et al., 2005b; Zhou and Guo, 2009). Three gamma-ray-irradiated mutants with increased chilling tolerance have been selected. The mutants maintained higher antioxidant activities and higher net photosynthetic rate (*A*) during chilling (Lu et al., 2013). However, the molecular responses to chilling in *Stylosanthes guianensis* have not been revealed. In order to understand the mechanisms of chilling tolerance in *S. guianensis*, suppression subtractive hybridization (SSH) was conducted in the group to identify the differentially expressed genes in response to chilling between a chilling tolerant mutant 7–1 (Lu et al., 2013) and its wild type. One hundred and ninety-four sequences were harvested after confirmation by reversed Northern blot and the false positives were removed, and they have been subjected to a BlastX or BlastN comparison to the GenBank database online^1^. The largest group was classified as chloroplast and photosynthesis related genes including *CP12* (unpublished data), implying an association of the group of genes with chilling tolerance in *S. guianensis*.

A chloroplast protein CP12 has been found to regulate the Calvin cycle by forming a ternary complex with GAPDH and PRK, the key enzymes of the Calvin–Benson cycle (Graciet et al., 2004; Marri et al., 2009; Howard et al., 2011). The GAPDH/PRK/CP12 complex forms *in vivo* and results in a reduced activity of PRK and GAPDH under low light or in the dark, while it disassociates under light conditions and leads to an increased activity of GAPDH and PRK (Howard et al., 2008; López-Calcagno et al., 2014). GAPDH and PRK activities are inhibited by oxidants such as H_2_O_2_, oxidized glutathione (GSSG) and nitrosoglutathione (GSNO), while the GAPDH/CP12/PRK complexes are protected from treatments with oxidants in *Arabidopsis*. The results imply that CP12 protects GAPDH and PRK from oxidative stress (Marri et al., 2014). Three CP12 homologs, *CP12-1, CP12-2*, and *CP12-3*, are found in the genomes of *Arabidopsis*, rice and maize genome. Compared to CP12-3, CP12-1, and CP12-2 are highly homologous (López-Calcagno et al., 2014). The three *CP12* genes are differentially expressed in *Arabidopsis* (Marri et al., 2005; Singh et al., 2008). *CP12-2* expression depends upon light, which is similar to *GAPDH* and *PRK*, and is highest in photosynthetic tissues, while *CP12-1* is expressed in a range of tissues including photosynthetic tissues, root tips, flowers, and seeds. *CP12-3* is major expressed in roots, stigma and anthers (Singh et al., 2008). CP12 is required for plant growth and development. Carbon allocation to the cell wall and organic acids is increased and carbon going to starch and soluble carbohydrates is decreased in *CP12* antisense plants which showed a phenotype of dwarfism and reduced apical dominance (Howard et al., 2011). No severe photosynthetic or growth phenotype was observed in *Arabidopsis* single *cp12-1, cp12-2*, and *cp12-3* mutants, but reduced transcripts of both *CP12-1* and *CP12-2* lead to reduced PRK protein level and photosynthetic capacity and a phenotype with slower growth and reduced seed yield in double or triple mutants *CP12-1*/*2* and *CP12-1*/*2*/*3* (López-Calcagno et al., 2017). However, it is unknown what phenotypes display in transgenic plants overexpressing *CP12*. Response of *CP12* expression to low temperature and the role in chilling tolerance have not been reported.

The objectives of this study were to identify the differential expressed genes in response to chilling and to examine the role of *CP12* in chilling tolerance using transgenic *S. guianensis* plants overexpressing or down-regulating *CP12* expression.

## Materials and Methods

### Plant Materials and Treatments

Ten seedlings of the chilling-tolerant mutant 7–1, homozygous lines (T_3_) of transgenic *S*. *guianensis* plants overexpressing *SgCP12* or down-regulating *SgCP12* expression and the wild type (*S*. *guianensis* cv. CIAT184) were planted in 15-cm diameter plastic pots, respectively, containing mixture of peat and perlite (3:1, v/v), growing under natural light in a greenhouse at temperature from 25 to 30°C as previously described (Lu et al., 2013). For isolation of total RNA, one-month-old seedlings of 7–1 and the wild type plants were placed in a growth chamber for 2 d at 6°C with a 12-h photoperiod under light of 250 μmol m^-2^ s^-1^. For physiological measurements, two-month seedlings of transgenic plants and the wild type were placed in a growth chamber at 6°C for 8 d as described above, followed by sampling the second leaflets from the top. Then the plants were moved to room temperature for 1 week of recovery, and survival rate was measured. All experiments are replicated three times.

### Real-Time Quantitative Reverse Transcription PCR (qRT-PCR)

Total RNA was isolated from the second leaflets from the top at 0, 6, 24, and 48 h after chilling treatment, respectively, using TRIzol reagent (Invitrogen, Carlsbad, CA, United States). 1 μg of total RNA was used for synthesis of first-strand cDNA using the PrimeScript RT reagent Kit with gDNA Eraser (Takara, Shiga, Japan). PCR reaction mixture (10 μl) contained 15 ng of diluted cDNA template, 200 nM each of forward and reverse primers, and 5 μl SYBR Premix *Ex Taq* (Takara, Shiga, Japan). Parallel reactions to amplify *Actin1* were used to normalize the amount of template. The primers and their sequences are listed in **Supplementary Table S1**. qRT-PCR was conducted in Mini Option Real-Time PCR System (Bio-Rad, CA, United States) according to the manufacturer’s instructions. A negative control without cDNA was always included. Relative expression was calculated by 2^-ΔΔCt^, which was done automatically by the instrument. When compared the gene expression in transgenic plants or the mutant 7–1 with that in the wild type, relative expression was calculated based on defining gene expression in the wild type as one.

### Cloning of CP12 From *S. guianensis*

A 627 bp cDNA sequence of CP12 (FE192311.1) was recovered from the SSH cDNA library of *S. guianensis*. It contained the stop codon but lack a 45 bp fragment at the 5′-end. It is mostly homologous to pea *CP12* (CAA96570). For amplification of the remaining coding sequence of *SgCP12*, therefore, a forward primer ZG1129 (CAGAGGAAAAATGGCGACC) was designed based on the sequence of pea *CP12*, while the reverse primer ZG1130 (CTGATTATGAGGGCGGTTACA) was designed based on *S. guianensis CP12* cDNA (FE192311.1). PCR reaction mixture contained primers ZG1129 and ZG1130, the first-strand cDNA as the template, and *KOD*-*Plus* DNA polymerase (TOYOBO, Osaka, Japan). DNAMAN software (Lynnon Biosoft, Vaudreuil, Quebec, CA, Canada) was used for analysis of the deduced amino acid sequence of SgCP12.

### Generation of Transgenic Plants

The coding sequence of *SgCP12* was cloned to expression vector pCAMBIA3301 in sense or antisense direction under driven by CaMV 35S promoter. Cotyledons from 7-day-old seedlings was infected in suspension cells of *Agrobacterium tumefaciens* strain EHA105 harboring the expression construct, and transgenic *S. guianensis* plants were generated as described previously (Bao et al., 2016). One rooted plant from each Basta-resistant callus (independent transformant) was kept for transplanting to soil in plastic pots and grown in greenhouse.

### Evaluation of Plant Growth and Chilling Tolerance in Transgenic Plants

Transgenic *S. guianensis* plants were selected by spraying with 60 mg l^-1^ Basta herbicide at seedlings stage of T_0_, T_1_, and T_2_ plants to collect homozygous lines, and the survival plants were further confirmed by using PCR for detecting *bar* with primers ZG1409 (CAGCTGCCAGAAACCCACGT) and ZG1410 (CTGCACCATCGTCAACCACT). For evaluation of chilling tolerance, ion leakage and maximal photochemical efficiency of PSII (*F*_v_/*F*_m_) were measured after 8 d of chilling treatment at 6°C in growth chamber as described previously (Lu et al., 2013; Bao et al., 2016), while survival rate was measured based on recording the surviving plant numbers and total numbers in one pot after 7 d of recovery at room temperature post chilling treatment.

### Measurements of Net Photosynthetic Rate (Pn)

Net photosynthetic rate was measured as described previously (Zhou et al., 2006), using a LI-6400P Portable Photosynthesis System (LI-COR Inc., Lincoln, Nebraska, United States), by controlling leaf temperature at 25°C, CO_2_ concentration at 400 μmol l^-1^ and relative humidity at 70%.

### Measurements of Calvin–Benson Cycle Enzymes

Leaves were detached from plants with illumination for 2 h under light of 500 μmol m^-2^ s^-1^ and extracted in 1 ml of ice-cold extraction buffer containing 100 mM Tricine (pH 8.0), 10 mM MgCl_2_, 20 mM KCl, 1 mM EDTA, 5 mM DTT, and 0.05% Triton ×-100 (v/v), 5% glycerol (v/v), 1% PVP. The homogenates were centrifuged at 15,000 × *g* for 15 min, and the resulting supernatant was used for assay of PRK and GAPDH (Howard et al., 2011). PRK was assayed in reaction mixture containing 50 mM HEPES (pH7.8), 10 mM MgCl_2_, 40 mM KCl, 1 mM ATP, 4 mM DTT, 0.1 mM NADH, 2 units mL21 pyruvate kinase, 1 mM phosphenolpyruvate, 0.5 mM ribulose-5-phosphate, 2 units ml^-1^ lactate dehydrogenase, 2 units ml^-1^ pyruvate kinase, and the reaction was initiated by addition of the enzyme extract in a total reaction volume of 1 ml. GAPDH activity was assayed in reaction mixture containing 50 mM Tris–HCl (pH 7.8), 10 mM MgCl_2_, 1 mM EDTA, 2.5 mM ATP, 4 mM DTT, 0.1 mM NADPH, 5 mM 3-phosphoglyceric acid, 2 units ml^-1^ 3-phosphoglyceric phosphokinase and initiated by addition of the enzyme extract in a total reaction volume of 1 ml. Aldolase activity was assayed in reaction mixture containing 50 mM Tris–HC (pH 8.5), 1 mM EDTA, 5 mM MgCl_2_, 2 mM fructose-1,6-biphosphate (FBP), 0.15 mM NADH, 5 units ml^-1^ triosephosphate isomerase, 2 units ml^-1^ glycerol-3-phosphate dehydrogenase and initiated by addition of the enzyme extract in a total reaction volume of 1 ml. Fructose-1,6-bisphosphatase (FBPase) was assayed in reaction mixture containing 100 mM Tris–HCl (pH8.2), 5 mM MgCl_2_, 4 mM fructose-1,6-bisphosphate, 0.5 mM NADP, 2 units ml^-1^ phosphoglucose isomerase, 1 units ml^-1^ glucose-6-phosphate dehydrogenase, and the reaction was initiated by addition of the enzyme extract in a total reaction volume of 1 ml.

### Statistical Analysis

All data were subjected to analysis of variances (ANOVA) according to the model for completely randomized design using an SPSS program (SPSS Inc, Chicago, IL, United States). Differences among means of plant lines were evaluated by Duncan test at 0.05 probability level.

## Results

### Transcripts of the Differentially Expressed Genes in Response to Chilling in a Chilling-Tolerant Mutant as Compared With the Wild Type

**FIGURE 1:**
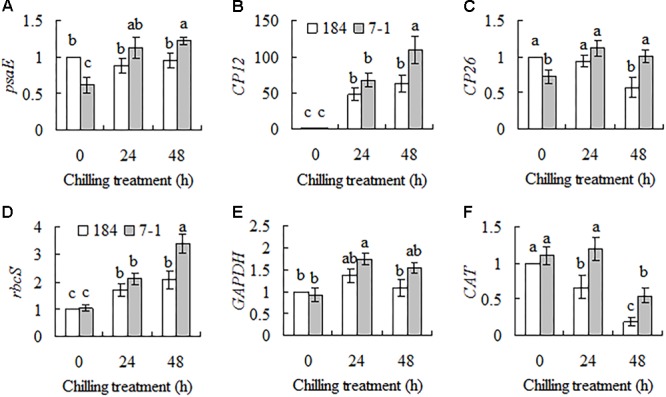
Analysis of transcript levels of six genes, *psaE* **(A)**, *CP12* **(B)**, *CP26* **(C)**, *rbcS* **(D)**, *GAPDH* **(E)**, and *CAT* **(F)**, in response to chilling stress between the chilling tolerant mutant 7–1 and the wild type (*S. guianensis* cv. CIAT184). Plants were placed in a growth chamber at 6°C for chilling treatment. *Actin* was used as reference gene for quantitative RT-PCR. Means of three replicates and standard errors are presented; the same letter above the column indicates no significant difference among the tested plant lines at *P* < 0.05.

Eight genes showing differential expression between a chilling tolerant mutant 7–1 and the wild type in SSH library were selected for confirmation, and a time course of transcripts in response to chilling stress was measured using qRT-PCR. Five genes are involved in photosynthesis, including *psaE* (encoding photosystem I reaction center subunit psaE), *CP12, CP26* (encoding chloroplast protein 26), *rbcS* (encoding Rubisco small subunit) and *GAPDH*, and the other one *CAT* (encoding catalase) is stress related. Compared to unaltered *psaE* transcript in the wild type, continuous induction in *psaE* transcript was observed in mutant 7–1 during chilling stress (**Figure 1A**). *SgCP12* transcript was continuously and greatly induced in both 7–1 and the wild type during chilling treatment, and higher level was observed in 7–1 than in the wild type after 48 h of chilling (**Figure 1B**). *CP26* transcript was decreased in the wild type after 48 h of chilling treatment, but it was induced in 7–1 after 24 h of chilling (**Figure 1C**). Rubisco is consisted of four large subunits (rbcL) and four small subunits (rbcS) and catalyzes fixation of CO_2_ by ribulose-1,5-bisphosphate (RuBP). *rbcS* transcript was induced continuously in both 7–1 and the wild type, and higher level was observed in 7–1 after 48 h of chilling (**Figure 1D**). *GAPDH* transcript was not altered significantly in the wild type during chilling treatment, while that was slightly induced in 7–1 at 24 h after chilling (**Figure 1E**). CAT transcript was continuously reduced in the wild type after chilling treatment, while it was unaltered in 7–1 at 24 h and decreased at 48 h after chilling. Higher level of CAT transcript was maintained in 7–1 as compared with the wild type at 24 and 48 h after chilling (**Figure 1F**).

### Cloning and Sequence Analysis of *SgCP12*

**FIGURE 2:**
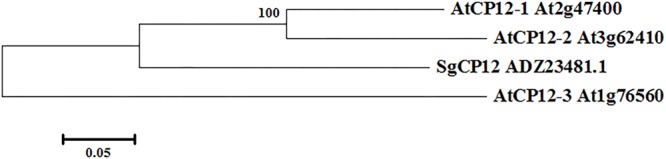
Phylogenetic relationship between SgCP12 and AtCP12 members in *Arabidopsis thaliana*. The phylogenetic tree was constructed with the DNAMAN program. The bar represents the branch length equivalent to 0.05 amino acid changes per residue.

Given that *SgCP12* transcript was greatly induced in response to chilling stress and showed difference between 7–1 and the wild type, it implies that *SgCP12* expression might be associated with chilling tolerance. Thus the role of *SgCP12* in chilling tolerance was investigated in the present study. The coding sequence of *SgCP12* was cloned from *S. guianensis*: it contains a 399 bp ORF (HQ906668) encoding a putative polypeptide of 132 amino acids (ADZ23481.1). A chloroplast transit peptide exists at the N-terminal region, indicating SgCP12 might be located on chloroplast. SgCP12 is most homologous to PsCP12 in pea (CAA96570) with 65% identification in amino acid sequence. Phylogenic analysis of SgCP12 with CP12 family in *Arabidopsis* showed that SgCP12 was more similar to AtCP12-2 or ATCP12-1 than AtCP2-3 (**Figure 2**).

### Analysis of Transgenic *S. guianensis* Plants

**FIGURE 3:**
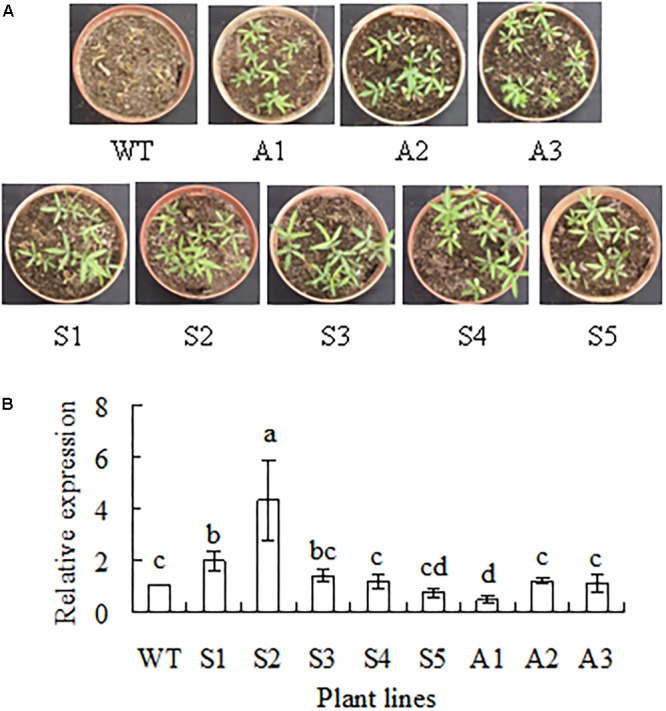
Basta resistance and relative expression of *SgCP12* in transgenic plants overexpressing *SgCP12* or down-regulating *SgCP12* expression in comparison with the wild type (*S*. *guianensis* cv. CIAT184). Two-week-old seedlings were sprayed with basta (60 mg l^-1^) to select transgenic plants, and photography was taken 1 week after spraying with basta **(A)**. Relative expression was measured using quantitative RT-PCR, and *actin* was used as reference gene **(B)**. Means of three replicates and standard errors are presented; the same letter above the column indicates no significant difference among the tested plant lines at *P* < 0.05.

Transgenic plants expressing *SgCP12* driven by CaMV35S promoter and down-regulating *SgCP12* expression by antisense approach were generated from individual Basta-resistant callus using *bar* as selective marker gene. Basta resistance was used to select transgenic plants from T_0_, T_1_, and T_2_ plants, respectively. For example, 75% of T_1_ transgenic plants were survival, while all the wild type plants were dead after spraying with Basta (**Figure 3A**). Homozygous lines with resistance to Basta were selected from T_2_ plants. Among the five lines overexpressing *SgCP12* lines S1 and S2 had significantly increased *SgCP12* transcript; among three antisense lines A1 had decreased *SgCP12* transcript, while in A2 and A3 the transcript level was not altered (**Figure 3B**). Thus, S1, S2 and A1 were used for further investigation.

### *SgCP12* Expression Affected Plant Growth and Chilling Tolerance

**FIGURE 4:**
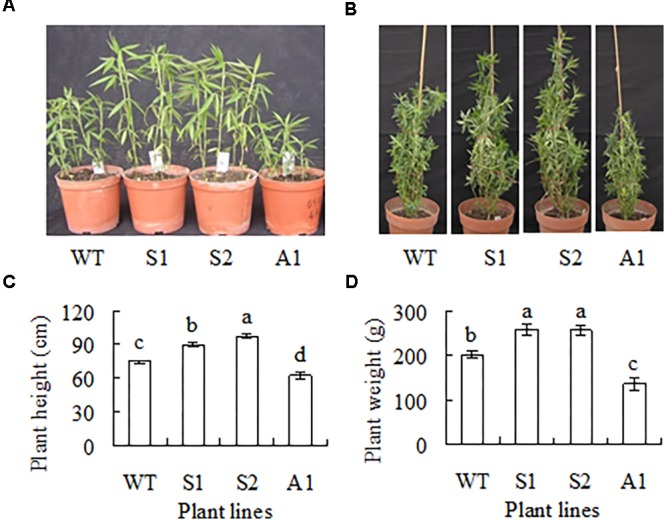
Phenotype of transgenic plants overexpressing *SgCP12* or down-regulating *SgCP12* expression in comparison with the wild type (*S*. *guianensis* cv. CIAT184). The photos show five-week-old **(A)** and 12-week-old plants **(B)**. Plant height **(C)** and fresh weight per plant above ground **(D)** were measured 12 weeks after planting. Means of data from ten plants and standard errors are presented; the same letter above the column indicates no significant difference among the tested plant lines at *P* < 0.05.

Plant growth showed a difference between transgenic plants and the wild type after 5 and 12 weeks growth in greenhouse (**Figures 4A,B**). Plant height was increased by 20 and 30%, and biomass was increased by 29 and 27%, respectively, in lines S1 and S2 as compared with the wild type, while in A1 plant height and biomass were decreased by 17 and 32%, respectively (**Figures 4C,D**). Lines S3, S4, S5, A2, A3, and 184 showed no difference in plant height and fresh weight of shoots (data not shown). The results indicated that up-regulation of *SgCP12* expression resulted in promoted growth, while down-regulation led to slow growth.

**FIGURE 5:**
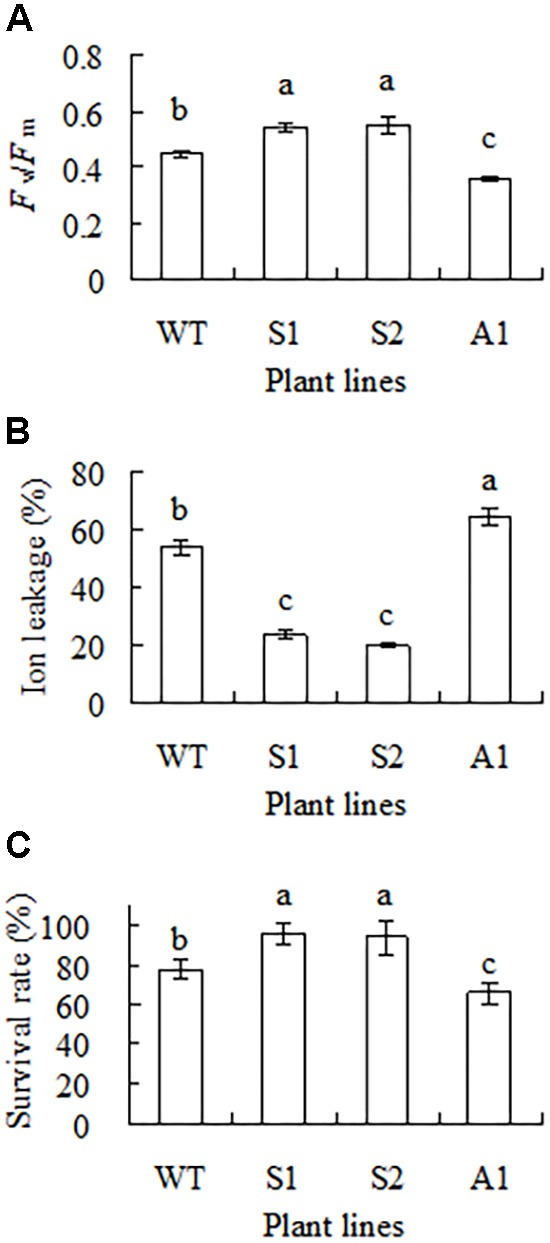
Analysis of chilling tolerance in transgenic plants overexpressing *SgCP12* or down-regulating *SgCP12* expression in comparison with the wild type (*S*. *guianensis* cv. CIAT184). *F*_v_/*F*_m_ **(A)** and ion leakage **(B)** were measured 8 d after chilling treatment at 6°C, while survival rate **(C)** was measured after 7 d of recovery at room temperature post chilling treatment. Means of three replicates and standard errors are presented; the same letter above the column indicates no significant difference at *P* < 0.05.

Chilling tolerance was assessed based on measurements of *F*_v_/*F*_m_, ion leakage and survival rate. Compared to the wild type, higher *F*_v_/*F*_m_ and lower ion leakage was observed in S1 and S2; lower *F*_v_/*F*_m_ and higher ion leakage was observed in A1 (**Figures 5A,B**). After 1 week of recovery at room temperature, 22 and 34% of the plants were dead in the wild type and line A1, respectively, while 95% of the transgenic plants were survival (**Figure 5C**). The results indicated that up-regulation of *SgCP12* expression resulted in increased chilling tolerance, while down-regulation resulted in decreased chilling tolerance.

### *SgCP12* Expression Affected Net Photosynthetic Rate and Activities of PFK and GAPDH

**FIGURE 6:**
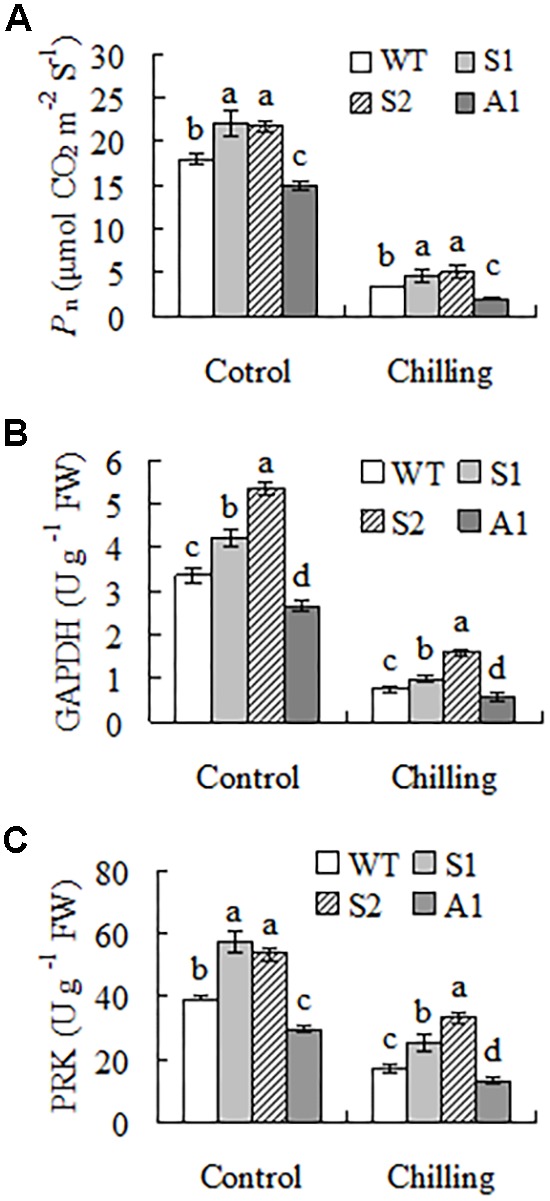
Net photosynthetic rate (*P*_n_) and enzyme activities of carbon assimilation in transgenic plants overexpressing *SgCP12* or down-regulating *SgCP12* expression in comparison with the wild type (*S*. *guianensis* cv. CIAT184). *P*_n_ **(A)** and activities of GAPDH **(B)** and PRK **(C)** were measured before chilling treatment or 8 d after chilling treatment at 6°C. Means of three replicates and standard errors are presented; the same letter above the column indicates no significant difference at *P* < 0.05.

Compared to the wild type, lines S1 and S2 had higher *P*_n_ (21 to 23%) and line A1 had lower *P*_n_ (17%) under regular growth condition. Chilling temperature resulted in greatly decreased *P*_n_ in all plants, but higher levels were maintained in lines S1 and S2 (37 to 51%) and lower level (41%) was observed in line A1 (**Figure 6A**). Activities of GAPDH and PRK, like *P*_n_, were higher in lines S1 and S2 and lower in line A1 as compared with the wild type under control condition. Chilling inhibited the enzyme activities in all plants, but higher activities of GAPDH and PRK were observed in lines S1 and S2 and lower activities of GAPDH and PRK were observed in line A1 (**Figures 6B,C**). Aldolase and FBPase activities were decreased in all plants, but showed no difference between the wild type and transgenic plants overexpressing or down-regulating *SgCP12* (data not shown).

## Discussion

psaE is one of components of PSI, whiel CP26 is one of the minor antenna complexes of photosystem II (PSII) (Dekker and Boekema, 2005). *rbcS* encodes the small subunit of Rubisco that functions to catalyzes fixation of CO_2_ using ribulose-1,5-bisphosphate (RuBP) as substrate. Our results showed that, compared to the wild type, transcripts of *psaE, CP26, rbcS* and *GAPDH* were induced in the mutant 7–1, and higher levels were maintained in 7–1 than in the wild type during chilling stress. This is consistent with our previous observation that 7–1 had higher levels of PSII activity and *P*_n_ than the wild type (Lu et al., 2013). In addition, higher *CAT2* transcript was maintained in 7–1 than in the wild type during chilling, which is consistent with that higher CAT activity was observed in 7–1 than in the wild type under chilling stress (Lu et al., 2013). The results suggest that the increased capacity to use the absorbed light energy for photochemical reactions and the Calvin–Benson cycle for protection of oxidative damages under chilling stress is associated with the chilling tolerance in 7–1. The mutant 7–1 was produced by gamma-ray irradiation (Lu et al., 2013). Deletions of DNA fragment and base substitutions are frequently observed in gamma-ray induced mutants (Yoshihara et al., 2013), which might be associated with the altered gene expression in response to chilling in 7–1 due to mutations in some transcription factors or in the promoter regions of the genes.

*SgCP12* transcript was greatly induced in both 7–1 and the wild type with higher level in 7–1, implying a potential role of *SgCP12* in chilling tolerance in *S. guianensis*. The assumption was confirmed by transgenic plants. Overexpression of *SgCP12* resulted in enhanced chilling tolerance with higher survival rate and *F*_v_/*F*_m_ and lower ion leakage in transgenic plants as compared with the wild type, while down-regulation of *SgCP12* led to a reduced chilling tolerance with lower survival rate and *F*_v_/*F*_m_ and higher ion leakage. Our results suggest that *SgCP12* expression regulates chilling tolerance in *S. guianensis*. This is the first report to validate the role of *SgCP12* in chilling tolerance. In addition, *SgCP12* expression influenced plant growth. Up-regulation of *SgCP12* expression resulted in promoted plant growth with increased plant height and biomass, while down-regulation led to reduced plant height and biomass. The phenotype in antisense *S. guianensis* are similar to *CP12* antisense transgenic tobacco and *Arabidopsis* double or triple mutants *cp12-1*/*2* and *cp12-1*/*2*/*3* showing a reduced growth phenotype (Howard et al., 2011; López-Calcagno et al., 2017). It is suggested that *SgCP12* is a good candidate gene used for improving plant growth and chilling tolerance.

The Calvin–Benson cycle provides plants with carbohydrates and the intermediates used for synthesis of other organic substances. Rubisco plays a key role in CO_2_ fixation using RuBP as substrates for production of 3-phosphoglycerate (3-PGA), which can be further conversed to GAP, catalyzed by GAPDH. Except for one PGA being used for biosynthesis of carbohydrates, five PAG are used for regeneration of RuBP, in which several enzymes including aldolase, FBPase, and PRK are involved. PRK catalyzes the last step of RuBP regeneration (Michelet et al., 2013). There was no difference in aldolase and FBPase activities between the wild type and transgenic plants overexpressing or down-regulating SgCP12. PRK and GAPDH activities were lower in antisense plants than in the wild type under both control and chilling conditions. Likely, reduced PRK protein levels were observed in *Arabidopsis* mutants lacking *CP12-1* transcript, but Rubisco and GAPDH protein levels were not altered (López-Calcagno et al., 2017). There was a slight reduction in GAPDH and PRK activities in *CP12* antisense tobacco plants although it is not statistically different (Howard et al., 2011). In addition, higher activities of GAPDH and PRK were observed in *SgCP12* overexpression transgenic lines. The higher or lower PRK activity may promote or decrease RuBP regeneration and therefore increase or decrease CO_2_ assimilation in transgenic plants overexpressing or down-regulating *SgCP12*, respectively. Our results suggest that the altered activities of GAPDH and PRK are associated with the changes in *P*_n_ in transgenic *S. guianensis* overexpressing or down-regulating SgCP12, which in turn is associated with the phenotypes of transgenic plants. Nevertheless, the enhanced activities of the Calvin–Benson cycle enzymes promoted the usage of absorbed light energy for avoiding the oxidative damages induced by chilling in transgenic *S. guianensis* overexpressing *SgCP12*.

## Author Contributions

KL, HQ, MZ, and YL conducted the experiments. ZG and SL designed the experiments and wrote the manuscript.

## Conflict of Interest Statement

The authors declare that the research was conducted in the absence of any commercial or financial relationships that could be construed as a potential conflict of interest.

## Footnotes

1 http://www.ncbi.nlm.nih.gov/
