# Supplementary material for: Chloroplast Protein 12 Expression Alters Growth and Chilling Tolerance in Tropical Forage Stylosanthes guianensis (Aublet) Sw
Source: Front Plant Sci. 2018 Sep 6;9:1319. doi: 10.3389/fpls.2018.01319 (PMC6135879; doi:10.3389/fpls.2018.01319)
Supplement: Supplementary file 1 [file Table_1.docx]

**Table S1** Primers used for real-time quantitative reverse transcription PCR (qRT-PCR)

| Gene name | Accession No. | Primer name | Sequence |
| --- | --- | --- | --- |
| *psaE* | FE192328 | ZG2209 | 5’-GCATTGGATGAGATTGTAGA-3’ |
|  |  | ZG2210 | 5’-AGATCAGACGTTGATGGATA-3’ |
| *CP12* | HQ906668 | ZG1773 | 5’-TAGTCAGGCTCAACCAGA-3’ |
|  |  | ZG1774 | 5’-CGCTTATGCTCTTCTCTACC-3’ |
| *CP26* | FE192392 | ZG2189 | 5’-AGGAGATCAAGAATGGAAGG-3’ |
|  |  | ZG2190 | 5’-AGAACCAGCAATGACAGT-3’ |
| *rbcS* | FE192378 | ZG2201 | 5’-TTGTCTACCGTGAGAACC-3’ |
|  |  | ZG2202 | 5’-TTAATGACCTGAGCAGAG-3’ |
| *GAPDH* | FE192388 | ZG2191 | 5’-TGATGACATGGTGAAGGT-3’ |
|  |  | ZG2192 | 5’-AGAAGTGTAGTGGTAGTAGTAG-3’ |
| *CAT2* | FE192344 | ZG2203 | 5’-GGTCAGAAGGTAGCAAGT-3’ |
|  |  | ZG2204 | 5’-GTATAACAGAGGAGTCACATC-3’ |
| *Actin* | MH270639 | ZG1781 | 5’-GTTCTTCTCCAGCCATCT-3’ |
|  |  | ZG1782 | 5’-CCTTGCTCATACGGTCAG-3’ |
